# Characterization of hippocampal sclerosis of aging and its association with other neuropathologic changes and cognitive deficits in the oldest-old

**DOI:** 10.1007/s00401-023-02606-9

**Published:** 2023-06-29

**Authors:** Lorena Sordo, Tianchen Qian, Syed A. Bukhari, Katelynn M. Nguyen, Davis C. Woodworth, Elizabeth Head, Claudia H. Kawas, María M. Corrada, Thomas J. Montine, S. Ahmad Sajjadi

**Affiliations:** 1 Department of Neurology, University of California, Irvine, Office 364, Med Surge II Building, Irvine, CA 92697 USA; 2 Institute for Memory Impairments and Neurological Disorders, University of California, Irvine, CA USA; 3 Department of Pathology and Laboratory Medicine, University of California, Irvine, CA USA; 4 Department of Statistics, University of California, Irvine, CA USA; 5 Department of Pathology, School of Medicine, Stanford University, Palo Alto, CA USA; 6 Department of Neurobiology and Behavior, University of California, Irvine, CA USA; 7 Department of Epidemiology and Biostatistics, University of California, Irvine, CA USA

**Keywords:** Hippocampal subfields, Cognitive impairment, Quantification, Oldest-old, Arteriolosclerosis, LATE-NC

## Abstract

Hippocampal sclerosis of aging (HS-A) is a common age-related neuropathological lesion characterized by neuronal loss and astrogliosis in subiculum and CA1 subfield of hippocampus. HS-A is associated with cognitive decline that mimics Alzheimer’s disease. Pathological diagnosis of HS-A is traditionally binary based on presence/absence of the lesion. We compared this traditional measure against our novel quantitative measure for studying the relationship between HS-A and other neuropathologies and cognitive impairment. We included 409 participants from *The 90*+ *study* with neuropathological examination and longitudinal neuropsychological assessments. In those with HS-A, we examined digitized H&E and LFB stained hippocampal slides. The length of HS-A in each subfield of hippocampus and subiculum, each further divided into three subregions, was measured using Aperio eSlide Manager. For each subregion, the proportion affected by HS-A was calculated. Using regression models, both traditional/binary and quantitative measures were used to study the relationship between HS-A and other neuropathological changes and cognitive outcomes. HS-A was present in 48 (12%) of participants and was always focal, primarily affecting CA1 (73%), followed by subiculum (9%); overlapping pathology (subiculum and CA1) affected 18% of individuals. HS-A was more common in the left (82%) than the right (25%) hemisphere and was bilateral in 7% of participants. HS-A traditional/binary assessment was associated with limbic-predominant age-related TDP-43 encephalopathy (LATE-NC; OR = 3.45, *p* < 0.001) and aging-related tau astrogliopathy (ARTAG; OR = 2.72, *p* = 0.008). In contrast, our quantitative approach showed associations between the proportion of HS-A (CA1/subiculum/combined) and LATE-NC (*p* = 0.001) and arteriolosclerosis (*p* = 0.005). While traditional binary assessment of HS-A was associated with impaired memory (OR = 2.60, *p* = 0.007), calculations (OR = 2.16, *p* = 0.027), and orientation (OR = 3.56, *p* < 0.001), our quantitative approach revealed additional associations with impairments in language (OR = 1.33, *p* = 0.018) and visuospatial domains (OR = 1.37, *p* = 0.006). Our novel quantitative method revealed associations between HS-A and vascular pathologies and impairment in cognitive domains that were not detected using traditional/binary measures.

## Introduction

Hippocampal sclerosis of aging (HS-A) is a degenerative pathology characterized by loss of neurons and astrogliosis in the subiculum and cornu ammonis (CA1) subregion of the hippocampus [3]. HS-A is strongly associated with dementia in the oldest-old [29] and clinically, it mimics Alzheimer’s disease. Previous studies have reported a very wide range of HS-A prevalence from as low as 0.4% to as high as 26% [2, 17, 30, 31, 38, 39, 45, 60]. This wide range in prevalence is likely multifactorial due to differences in the cohorts assessed (e.g., age) as well as differences in methodologies, sampling methods, and HS-A diagnostic criteria [2, 6, 17, 31, 59, 60]. HS-A can be complete, when it affects the entire extent of the hippocampus, or focal. It also can affect the hippocampus unilaterally or bilaterally. Even though HS-A is a very important neuropathological change in aged individuals, there is paucity of data on its quantitative extent (focal vs. complete) and laterality [24, 30, 31, 47, 60], factors that might have caused the significant variability in its reported prevalence and cognitive impact.

HS-A is typically accompanied by other neurodegenerative diseases, including pathologic TDP-43 neuronal inclusions, Alzheimer disease neuropathologic change (ADNP), vascular disease, brain injury, and frontotemporal lobar degeneration [4–6, 10, 17, 22, 24, 38, 45, 60]. The association between HS-A and pathologic TDP-43 inclusions is well established but previous studies have reported conflicting results on the association of HS-A and other pathologic features [4, 24, 38, 60].

In this study, we pursued three aims: (1) using a novel quantitative method, we established the prevalence and quantitative distribution of HS-A in subiculum and different subfields of the hippocampus in the oldest-old. We then used these quantitative measures of HS-A distribution to, (2) examine the relationship between the presence and extent of HS-A and other neuropathological changes, and (3) examine the relationship between HS-A and dementia and impairment in cognitive domains.

## Materials and methods

### Participants

Participants from *The 90*+ *study* who had agreed to longitudinal in-person assessments and *post-mortem* brain examination were included. *The 90*+ *study* is a community-based cohort in Southern California, USA, that was developed to study the physical and mental health of the oldest-old, the fastest growing age group in the United States [15]. Initially, participants in *The 90*+ *study* were survivors from the Leisure World Cohort Study (LWCS), an epidemiologic investigation of a retirement community in Orange County, CA in the early 1980s [43, 44]. *The 90*+ *study* was initiated in 2003 when the surviving participants from the original LWCS who were aged 90 years and older on January 1, 2003, were invited to join***.*** A similar invitation was extended by *The 90*+ *study* on January 1, 2008, and every year thereafter to those turning 90 years of age. More recently, volunteers aged 90 and older beyond the LWCS are recruited using a variety of methods including mailing lists of residences believed to have people aged 90 and older, talks at local communities, talks to primary doctors, ads in local newspapers, and relatives or friends of participants. Participants from the LWCS were recruited regardless of cognitive diagnosis, whereas non-LWCS volunteers had no or mild dementia. The inclusion criteria for the present study were availability of (1) brain autopsy results and (2) clinical diagnosis from a multidisciplinary *post-mortem* case-conference and neuropsychological data.

### Standard protocol approvals, registrations, and patient consents

All participants or their designated surrogates provided consent to participate in the study. Procedures were reviewed and approved by the Institutional Review Board at the University of California, Irvine.

### Assessments

Participants underwent evaluations every 6 months. Clinical evaluations included a battery of neuropsychological tests, neurological exam, and self or informant completed questionnaires for demographics and medical history. Furthermore, after a participant’s death, additional information was obtained from informants regarding cognition and function since the last evaluation. Also, medical history information from the obtained medical records were incorporated to self- or informant-provided medical history.

### Medical history variables

Medical history variables were obtained from participants or their informant at the first visit and were updated at every follow-up visit. Medical history variables included hypertension (HTN), hypercholesterolemia, heart disease, chronic obstructive pulmonary disease (COPD), diabetes, stroke, transient ischemic attack (TIA), seizure, rheumatological disease, osteoarthritis, macular degeneration, glaucoma, thyroid disease, depression, and anxiety. Heart disease and rheumatological illness were composite variables. Heart disease was considered present if the patient had a history of any of coronary artery disease, atrial fibrillation and other arrhythmia, congestive heart failure, myocardial infarction, heart valve disease, coronary artery bypass and pacemakers. Rheumatological disease history was established based on the answer participants gave to a question asking if they had history of any of lupus, scleroderma, or rheumatoid arthritis.

### Determination of dementia and impaired cognitive domains

Clinical diagnosis of dementia was made applying Diagnostic and Statistical Manual of Mental Disorders 4th edition (DSM-IV) criteria in a multidisciplinary consensus diagnostic conference done after a participant’s death, with conferees blinded to pathological findings. All available information on the participant was used in determination of dementia. This included neuropsychological test scores, neurological examination, information collected from informants, available medical records, videos containing semi-structured interviews about their daily life and questions to test their memory, as well as brief examination of their gait from the time of visits, and death certificates. Similarly, identification of the impaired cognitive domains was based on consideration of participants’ scores in longitudinal neuropsychological tests, information obtained from their informants, and their conduct in recorded semi-structured interviews that were reviewed at *post-mortem* conferences. Specifically, determination of memory impairment is based on participants’ longitudinal performance in the short (nine items) version of California Verbal Learning Test (CVLT), their performance in memory sub-domains of mini-mental state examination (MMSE), and modified mini-mental state examination (3MS), and their conduct in the semi-structured interviews. Language impairment is diagnosed based on the performance in the 15-item version of Boston naming test, category fluency, and performance in semi-structured interviews. Visuospatial function is mainly assigned based on systematically collected information about participants’ problems with finding their way indoors or outdoors. Orientation is considered impaired when participants struggle with the temporal and spatial orientation items from MMSE. Specifically, participants are asked questions about date, day of the week, and season (temporal orientation) and the address of the place of assessment to include state, county, city, building, and floor (spatial). Finally, executive function impairment is diagnosed when there is abnormal performance in Trails-B and clock drawing tests and reduced letter fluency or, when there is informant report of problems with complex tasks. Performance in neuropsychological tests is typically depicted against standard norms of age-matched counterparts [57] to aid determination of whether the performance is within the normal range at each visit.

### Pathological evaluations

All *post-mortem* brain procurements were performed at the University of California (UC), Irvine between 2003 and 2021 using Alzheimer’s Disease Research Center (ADRC) protocols. Before dissection, the whole brain was weighed and then one hemisphere was selected based on the clinician’s impression of any asymmetry in clinical features. In most cases, the left hemisphere was the primary hemisphere (*n* = 35), and the right hemisphere was the primary hemisphere in the remaining cases (*n* = 9). The selected hemisphere, contralateral hippocampus, and contralateral cerebellum with brainstem were fixed with 4% paraformaldehyde for at least 2 weeks before dissection. Beginning in January 2017, all pathological evaluations have been performed at Stanford University using state-of-the-art neuropathological methods according to current NIA‐AA neuropathologic guidelines [25, 35]. To unify the pathological evaluations across the samples procured over two decades, all brains procured before 2017 were shipped to Stanford University and were re-sampled, re-sectioned, and re-evaluated, followed by histochemical and immunohistochemical stains exactly according to the protocols used in cases collected after 2016. We considered the following pathologic changes for analysis: HS-A, Alzheimer’s disease neuropathologic change (ADNC), limbic-predominant age-related TDP-43 encephalopathy neuropathologic change (LATE-NC), Lewy bodies (LBD), arteriolosclerosis, atherosclerosis, microvascular lesions (MVL), cerebral amyloid angiopathy (CAA), and aging-related tau astrogliopathy (ARTAG). Arteriolosclerosis was defined as the hyaline thickening of the vessel walls commonly found in deep penetrating small vessels of the brain with smooth muscle degeneration and fibrohyalinotic thickening resulting in a narrow vascular lumen. Scoring of arteriolosclerosis consisted in 0 = normal small blood vessels; 1 = mild thickening of the vessel walls with mild fibrosis; 2 = partial loss of smooth muscle in the media with moderate hyaline fibrosis (absence of smooth muscle cells necessary); 3 = complete loss of smooth muscle cells in the media, severe hyaline fibrosis, and presence of “onion-skin” type hyperplastic changes [11, 52]. The rest of the neuropathological lesions were defined, examined, and scored in standardized sections following NIA-AA guidelines [32, 35, 52, 54]. Sections examined for arteriolosclerosis and MVL included olfactory bulbs and tracks, middle frontal gyrus (MFG), inferior parietal lobule (IPL), superior and middle temporal gyri (SMTG), primary visual cortex (PVC), cingulate (anterior and posterior), striatum (with globus pallidus and nucleus basalis of Meynart), thalamus, amygdala, hippocampus, midbrain, pons, medulla (three levels including cervical cord, if present), and cerebellar cortex with dentate nucleus, as well as contralateral MFG, IPL, SMTG, PVC, striatum, thalamus, and hippocampus. For CAA, these regions included MFG, IPL, SMTG, striatum with GP and NB, hippocampus, midbrain and cerebellar cortex with dentate nucleus. For atherosclerosis, we examined the Circle of Willis and dorsal brain convexities. For ARTAG, we examined MFG, PVC, and hippocampus.

Of note, our oldest-old cohort did not include any individuals with FTLD-TDP or ALS-TDP neuropathologic changes.

### Hippocampal subfields and subregions and measurements

**Fig. 1:**
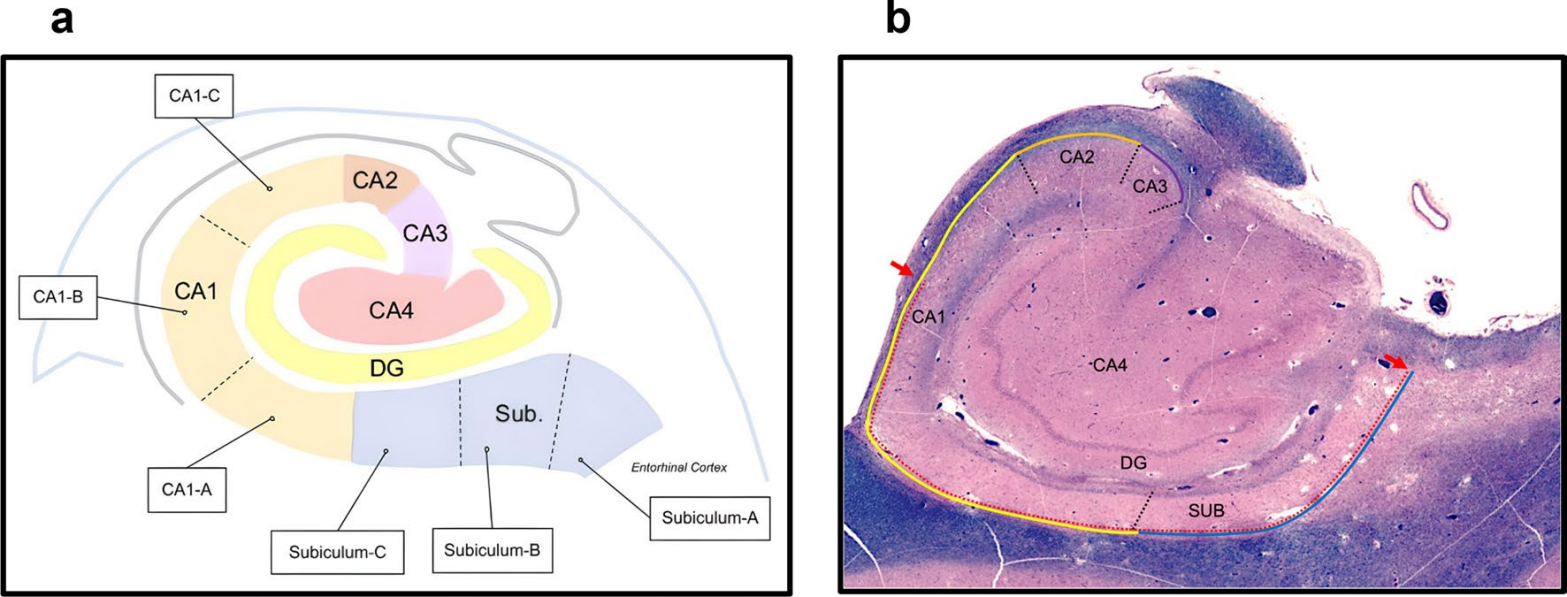
Hippocampal subfield, subregions, and measurements. **a** Each hippocampal subfield was partitioned into thirds to determine the different subregions. The end closest to the entorhinal cortex was labeled as “A”, the end closest to CA4 was labeled as “C”, and the region in between A and C was labeled as “B”. **b** For the quantitative measurements, the total length of each subfield (i.e., blue line for subiculum, yellow line for CA1, orange for CA2, and purple for CA3) was measured first. Then the length of the HS-A lesions was measured using the line annotation tool on Aperio eSlide Manager (dotted red lines). Using the total length of each subfield and subregion as denominator, we obtained ratios and reported the length of the HS-A lesions in proportions

Bilateral hippocampi were examined. Each hippocampus was dissected in the coronal plane to generate a section at the level of the lateral geniculate nucleus that included both entorhinal and transentorhinal cortices. All hippocampi were examined bilaterally at this level to avoid confounding factors. Hematoxylin and eosin (H&E) and Luxol fast blue (LFB) stained slides from individuals who received a pathological diagnosis of HS-A by traditional binary methods (i.e., presence/absence) were digitized with a Leica Aperio AT2 Scanner (Leica Biosystems) at 40× magnification. HS-A was defined as any extent of the neuropathological change involving a selective loss of pyramidal neurons in the hippocampal subfields and/or the subiculum that was accompanied by gliosis. Whole slide images were analyzed in an svs digital slide format using Aperio eSlide Manager (Leica Biosystems Imaging) web-based software. Once digitized, each of the hippocampal subfields (i.e., subiculum, CA1, CA2, and CA3) were delineated using the polygon annotation tool on eSlide Manager, and the total length of each subfield was measured using the line annotation tool. Subsequently, we performed a trisection of the subfield, where the arc length (i.e., outer length) of each subfield was divided into three equal portions (i.e., subregions) and labeled according to their proximity to entorhinal cortex (Fig. 1a). The subregion closest to the entorhinal cortex was labeled as “A”, the subregion closest to CA4 was labeled as “C”, and the subregion between A and C was labeled as “B” (e.g., subiculum-A, subiculum-B, and subiculum-C; CA1-A, CA1-B, and CA1-C).

To obtain quantitative measurements, the length of the HS-A lesion was measured using the line annotation tool. Ratios were obtained using the total length of each subfield as denominator and the length of the HS-A lesions was reported in proportions (Fig. 1b).

### Presence of other neuropathological changes and cognitive outcomes

Cognitive outcomes were established at the time of death and included presence of dementia and impairment in memory, language, executive function, visuospatial function, constructions, calculations, and orientation as determined at *post-mortem* conference. Presence of other pathological changes was determined by neuropathologic assessments following the current consensus NIA-AA guidelines [25, 35]. Severity of cerebral atherosclerosis and arteriolosclerosis were evaluated using methods previously published by us [8].

### Statistical analyses

We considered two types of measures for HS-A: traditional binary measure (presence/absence) and our novel quantitative measure (lengthwise proportion of HS-A in subiculum and/or hippocampal subfields). For traditional binary measure (presence of HS-A), we also examined the side of HS-A (left or right hemisphere). For novel quantitative measure, we considered side, subfield affected by HS-A (subiculum/CA1 or both), subregion of each subfield (see section “hippocampal subregions and measurements”), the proportion of HS-A in subregions of subiculum (subiculum-A/B/C), or CA1 (CA1-A/B/C), and the proportion of HS-A in subiculum and CA1 combined (defined as the total length of HS-A in the two subfields divided by the total length of the two subfields). Since quantitative HS-A data were missing for 4 individuals with HS-A, our quantitative analyses were performed using data from 44 of the total 48 individuals harboring HS-A. Other neuropathological changes were dichotomized as follows: ADNC was considered present in those with intermediate or high severity of ADNC according to NIA-AA guidelines [35]. LATE-NC was defined as present in those with involvement of either amygdala, hippocampus, or neocortex (stages 1, 2, or 3 according to the consensus paper [37]). Lewy body disease was considered present in those with involvement of limbic structures and/or neocortex in accordance with NIA-AA [35] and consensus guidelines [34]. MVLs were remote focal ischemic and/or hemorrhagic lesions found only on microscopic examination of standardized screening sections [55]. MVLs were scored positive if there were two or more MVLs in the screening sections [35]. Arteriolosclerosis and CAA were scored positive in those with any evidence of these pathologic changes (mild, moderate, or severe). Atherosclerosis was considered present only in those with severe atherosclerosis. ARTAG was scored positive in those with any ARTAG (occasional or numerous).

We first compared the characteristics between the groups (i) by testing whether demographic information and dementia at death differed in those with and without HS-A (Welch’s *t* test and Chi-squared test) (ii) to examine the presence of HS-A in each hemisphere and subfield (summary statistics) (iii) to test if quantitative HS-A measures in each subfield differed between left vs. right hemisphere (paired *t* test). We further conducted regression analyses to assess the association between HS-A and (iv) other neurodegenerative and vascular pathologies, (v) impairment of cognitive domains, and (vi) medical histories. All regressions were adjusted for age at death, sex, and education (college or above vs. otherwise). To answer whether HS-A was associated with other pathologic changes (iv), we considered the full cohort and examined the association of each of the neuropathological changes with outcomes of presence (logistic regression) and proportion in CA1/subiculum/combined regions (linear regression) of HS-A. To answer whether HS-A was related with impairment in cognitive domains (v), we fitted logistic regressions where, in each regression, the outcome was impairment in each cognitive domain and the regressors were one of the HS-A variables [whether someone has HS-A (binary measure), or the proportion of HS-A in subiculum/CA1 combined (quantitative measure)], using the full cohort. To answer the question whether HS-A was related to medical histories (vi), we used the same models as in question (iv) with medical history variables as the regressors.

Each analysis excluded individuals whose corresponding variables were missing. Heteroskedasticity-consistent standard errors were used in linear regression [56]. *p* values < 0.05 were considered statistically significant. All analyses were performed using R statistical software [48].

### Data availability

Data for all the analyses and results reported in this paper were acquired from *The 90*+ *study*. Data not published within the article will be shared by request from any qualified investigator.

## Results

### Demographics

The study sample comprised 409 individuals aged 90 years or older. Age at death ranged from 90.1 to 110.6 years old (mean 97.7). Most of the participants were female (69.4%) and half (50.6%) had a college degree or higher education (Table 1). HS-A was found in 48/409 (11.7%) of the cohort. Dementia at death was more prevalent among those with HS-A (66.7%) than those without HS-A (40.7%, *p* = 0.003). There were no significant differences in age at death, sex, or education between those with and without HS-A.

**Table 1:** Demographics in the full cohort and stratified by HS-A or the side affected by HS-A

	Full cohort *n* = 409	Without HS-A *n* = 361	With HS-A *n* = 48	*p* value	Right-sided HS-A *n* = 8	Left-sided HS-A *n* = 33	Bilateral HS-A *n* = 3
Age at death (years)				0.325			
Mean (SD)	97.7 (3.6)	97.6 (3.6)	98.1 (3.7)		100.7 (3)	97.9 (3.7)	97.3 (2.4)
Min, max	90.1, 110.6	90.1, 110.6	92.1, 106.2		97, 105.6	92.1, 106.2	95.1, 99.9
Sex				0.469			
Female	284 (69.4%)	248 (68.7%)	36 (75%)		6 (75%)	23 (69.7%)	3 (100%)
Male	125 (30.6%)	113 (31.3%)	12 (25%)		2 (25%)	10 (30.3%)	0 (0%)
Education				0.244			
Below college	202 (49.4%)	174 (48.2%)	28 (58.3%)		3 (37.5%)	20 (60.6%)	3 (100%)
College and above	207 (50.6%)	187 (51.8%)	20 (41.7%)		5 (62.5%)	13 (39.4%)	0 (0%)
Dementia at death				0.003			
Yes	178 (43.7%)	146 (40.7%)	32 (66.7%)		2 (25%)	25 (75.8%)	3 (100%)
No	229 (56.3%)	213 (59.3%)	16 (33.3%)		6 (75%)	8 (24.2%)	0 (0%)
Missing	2 (0.5%)	2 (0.6%)	0 (0%)		0 (0%)	0 (0%)	0 (0%)

*p* values were obtained from *t* tests (for age at death) or Chi-squared tests (for sex, education, and dementia at death) comparing those with HS-A and those without in the full cohort

### Hippocampal sclerosis of aging: side, subfield, and subregion distribution

**Fig. 2:**
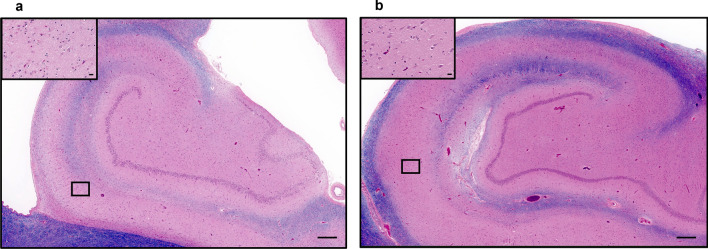
Hematoxylin and eosin (H&E) stained hippocampal slides from the same individual showing unilateral HS-A. **a** The left hippocampus harbored HS-A in subiculum and CA1 (insert), and **b** depicts no HS-A in any subfield of the right hippocampus. Scale bars of low magnification figures are 500 µm and 25 µm in inserts

In most of the cases, the presence and quantitative extent of HS-A lesions was assessed in all the subregions in both hemispheres. However, in 3 out of the 48 individuals, this was assessed unilaterally as one hippocampus was not available; in 2 of these individuals, the right hippocampus was assessed, whereas the left hippocampus was examined in 1 individual. None of the examined hippocampi harbored complete HS-A, defined as complete involvement of subiculum and CA1 region. Most of HS-A was unilateral (93%; 41/44 individuals; Fig. 2a and b) and the left hippocampus was affected in most HS-A cases (82%; 36/44 individuals), whereas the right hippocampus was affected in a quarter of the HS-A cases (25%; 11/44 individuals). Only 7% of the individuals harboring HS-A had it bilaterally (3/44) (Table 1).

**Fig. 3:**
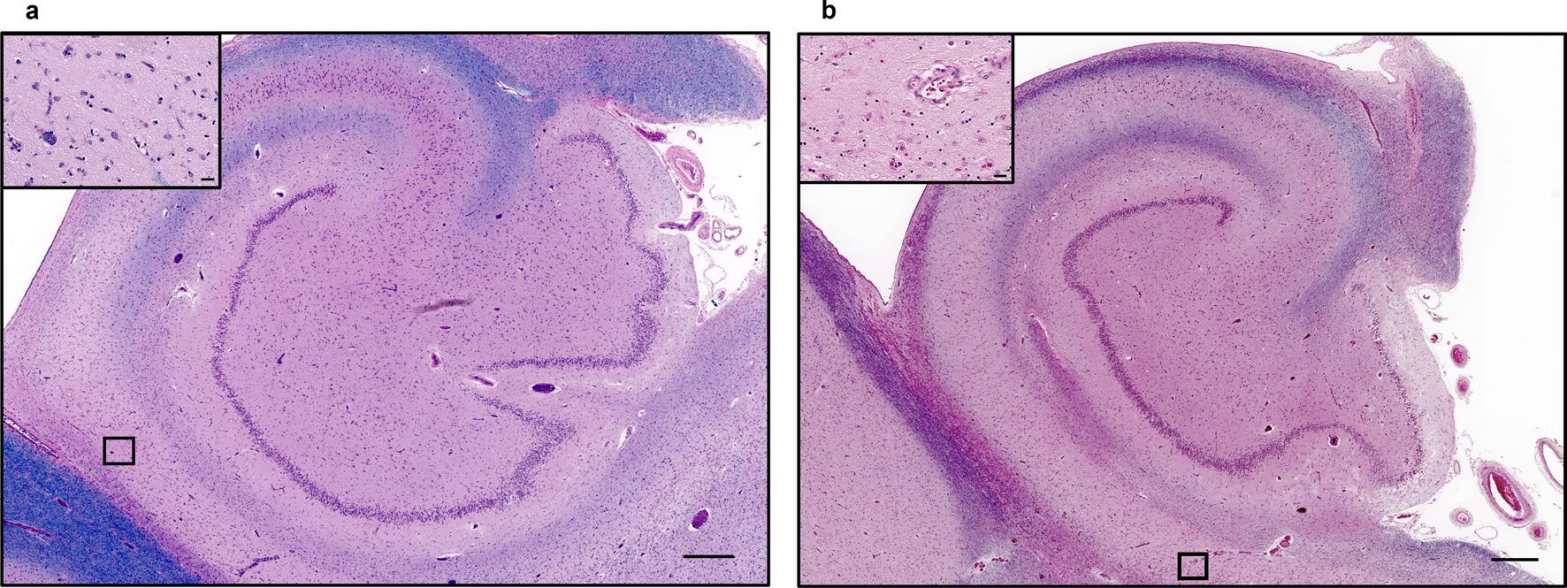
Hematoxylin and eosin (H&E) stained hippocampal slides from different individuals showing HS-A subfield involvement in **a** CA1 alone, and **b** subiculum alone. Scale bars of low magnification figures are 500 µm and 25 µm in inserts

**Fig. 4:**
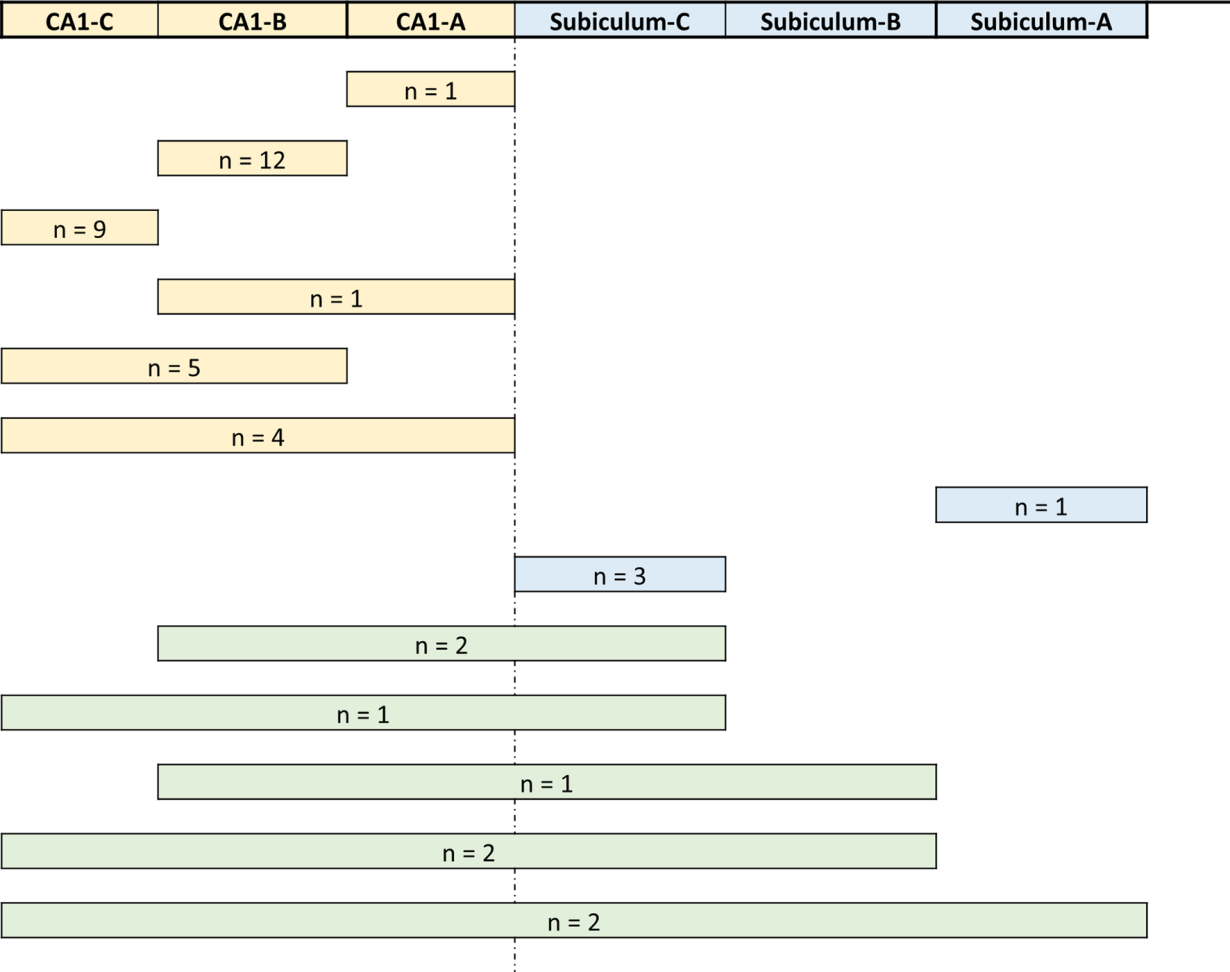
Distribution of hippocampal sclerosis of aging (HS-A) in the different subfields and subregions among the HS-A cohort (*n* = 44). HS-A most frequently affected CA1 alone (yellow; 32/44). In 8 out of the 44 individuals, HS-A was overlapping in both subiculum and CA1 (green). Involvement of subiculum alone (blue) was the least frequent (4/44). Since none of the examined hippocampi harbored complete HS-A, defined as complete involvement of subiculum and CA1 region, and spared some parts of each subregion, cases were considered to be focal HS-A. For individuals with symmetrical bilateral HS-A (*n* = 2), involvement of the relevant subregions is depicted; whereas for the individual with asymmetrical bilateral HS-A (*n* = 1), only the side with the most extensive involvement is depicted

In terms of subfield involvement, HS-A was far more frequent in CA1 alone (72.7%; 32/44 individuals; Fig. 3a) than subiculum alone (9.1%; 4/44; Fig. 3b), and none of the other hippocampal subfields were involved. In 18.2% of the individuals (8/44), there was overlapping HS-A in both subiculum and CA1. Notably, in most of these individuals, involvement of both subiculum and CA1 was focal and in only two (5%) participants, the whole subiculum was affected (Fig. 4). Among the 39 individuals with HS-A in CA1, 1 had involvement of CA1-A subregion only, 12 had CA1-B only involvement, and in 9, CA1-C was the only affected subregion. Four individuals had overlapping HS-A in both CA1-A and -B, and five in CA1-B and -C. Finally, while not involving the entirety of each subregion, eight individuals had overlapping involvement of all CA1 subregions. Given sparing of some parts of each subregion, these were still considered focal and not complete HS-A. Among the ten participants with focal HS-A in subiculum (i.e., not involving the full length of subiculum), one had involvement of subiculum-A only, six had involvement of subiculum-C only, and three had overlapping involvement of both subiculum-B and -C subregions.

Comparing the proportion of HS-A in each subregion by hemisphere affected, we found that on average, left hippocampus HS-A lesions were larger than right hippocampal HS-A. This difference between left and right lesions was statistically significant in CA1-A (*p* = 0.001), CA1-B (*p* = 0.044), and when considering both subiculum and CA1 combined (*p* = 0.002) (Table 2). There was also a trend for more extensive subiculum involvement (*p* ~ 0.1 for subiculum, subiculum-B and subiculum-C) for left-sided HS-A.

**Table 2:** Average proportions of hippocampal sclerosis of aging in each subregion stratified by hemisphere

	Average HS-A proportion (%) in right hippocampus	Average HS-A proportion (%) in left hippocampus	Difference in proportion (right − left)	*t* value	*df*	*p* value
Subiculum						
A	10.1	3.0	7.0	0.67	8	0.522
B	0.0	7.5	− 7.5	− 1.70	32	0.098
C	5.7	19.8	− 14.1	− 1.63	27	0.115
CA1						
A	0.0	27.6	− 27.6	− 3.59	31	0.001
B	18.4	40.0	− 21.5	− 2.12	24	0.044
C	7.7	17.3	− 9.6	− 1.37	12	0.195
Subiculum and CA1 combined	7.4	22.1	− 14.7	− 3.45	35	0.002

The first column represents the average of HS-A proportion in the right hemisphere and corresponding subregion, among those with right-sided only HS-A (*n* = 8). For example, the first entry 10.1 means that among those with right-sided only HS-A, the average HS-A proportion in subiculum-A of the right hemisphere is 10.1%. The second column is the average of HS-A proportion in the left hemisphere and corresponding subregion, among those with left-sided only HS-A (*n* = 33). Degrees of freedom (*df*) and *p* value are from Welch’s *t* test that accounts for unequal variances

Finally, of the three individuals with bilateral HS-A, one had bilateral HS-A in subiculum-B and -C, one had bilateral HS-A in CA1-C only, and the third had the most extensive lesion of the three: the left hippocampus of this individual was severely affected by HS-A, with lesions present in the whole subiculum (i.e., -A, -B, and -C) and in the whole CA1-A and -B. The subregion CA1-C of this individual was also partially affected. HS-A in the right hippocampus was present in subiculum-B and -C, and in CA1-B and -C. Interestingly, this individual with severe bilateral HS-A also had pathologic TDP-43 inclusions in amygdala, hippocampus, and cerebral cortical regions, whereas the other two individuals with less severe bilateral HS-A had pathologic TDP-43 lesions involving only amygdala and hippocampus.

### Hippocampal sclerosis of aging and other neuropathological changes

In the full cohort, the most common neuropathological change was arteriolosclerosis (83.4%), followed by ADNC (71.9%), ARTAG (61.4%), CAA (45%), LATE-NC (36.4%), LBD (15.4%), atherosclerosis (6.4%), and MVL (4.2%) (Table 3). The prevalence of co-occurring neuropathological changes in the HS-A cohort was similar to that in the full cohort with the exception of the frequency of LATE-NC that was nearly doubled in the HS-A cohort (64.6%).

**Table 3:** Co-occurrence of hippocampal sclerosis of aging and other neuropathological changes

	Full cohort *n* = 409	Without HS-A *n* = 361	With HS-A *n* = 48	Hemisphere affected			Subfield affected		
				Right only *n* = 8	Left only *n* = 33	Bilateral *n* = 3	Subic. only *n* = 4	CA1 only *n* = 32	Both *n* = 8
ADNC	294 (71.9%)	257 (71.2%)	37 (77.1%)	5 (62.5%)	26 (78.8%)	3 (100%)	3 (75%)	24 (75%)	7 (87.5%)
LATE-NC	149 (36.4%)	118 (32.7%)	31 (64.6%)	4 (50%)	21 (63.6%)	3 (100%)	2 (50%)	19 (59.4%)	7 (87.5%)
MVL	17 (4.2%)	15 (4.2%)	2 (4.2%)	0 (0%)	2 (6.1%)	0 (0%)	0 (0%)	2 (6.2%)	0 (0%)
LBD	63 (15.4%)	54 (15%)	9 (18.8%)	2 (25%)	7 (21.2%)	0 (0%)	0 (0%)	7 (21.9%)	2 (25%)
CAA	225 (55%)	193 (53.5%)	32 (66.7%)	3 (37.5%)	23 (69.7%)	3 (100%)	1 (25%)	22 (68.8%)	6 (75%)
Arteriolosclerosis	341 (83.4%)	299 (82.8%)	42 (87.5%)	7 (87.5%)	28 (84.8%)	3 (100%)	3 (75%)	27 (84.4%)	8 (100%)
Atherosclerosis	26 (6.4%)	21 (5.8%)	5 (10.6%)	0 (0%)	4 (12.1%)	0 (0%)	1 (20%)	1 (3.1%)	2 (33.3%)
ARTAG	251 (61.4%)	213 (59%)	38 (79.2%)	6 (75%)	26 (78.8%)	3 (100%)	3 (75%)	26 (81.2%)	6 (75%)

Atherosclerosis information was missing in three individuals in the full cohort; hence, the corresponding proportions were calculated after removing these individuals

In the full cohort, among these co-occurring neuropathological changes, LATE-NC and ARTAG were significantly associated with HS-A presence (binary measure). The odds of HS-A among individuals with LATE-NC was 3.81 times that among those without LATE-NC (95% CI 2.04–7.34, *p* < 0.001). The odds of HS-A among individuals with ARTAG was 2.72 times that among those without ARTAG (CI 1.35–5.98, *p* < 0.001) (Table 4). In contrast, when considering the quantitative measures of HS-A (proportions in subiculum, CA1, or both combined), significant associations with additional co-occurring neuropathological changes were identified (Table 4); however, the association with ARTAG was no longer significant. Arteriolosclerosis was associated with significantly increased HS-A proportions in subiculum, CA1, and subiculum and CA1 combined. MVL, on the other hand, was associated with significantly *decreased* HS-A proportion in subiculum only. LATE-NC still had the largest impact and was associated with significant increase in HS-A proportions in subiculum, CA1, and both subfields combined.

**Table 4:** Associations between binary or quantitative measures of HS-A and co-occurring neuropathological changes from logistic regression and linear regression on the full cohort

Regressor	Binary			Quantitative								
	HS-A presence			HS-A proportion in subiculum			HS-A proportion in CA1			HS-A proportion in subiculum and CA1		
	OR	95% CI	*p* value	beta	95% CI	*p* value	beta	95% CI	*p* value	beta	95% CI	*p* value
ADNC	1.28	(0.62, 2.62)	0.502	0.01	(− 0.01, 0.02)	0.201	0.01	(− 0.01, 0.03)	0.262	0.01	(− 0.01, 0.03)	0.217
LATE-NC	3.81	(2.02, 7.20)	< 0.001	0.03	(0.00, 0.05)	0.031	0.05	(0.02, 0.08)	0.001	0.04	(0.02, 0.07)	0.001
MVL	1.07	(0.23, 4.88)	0.932	− 0.01	(− 0.02, − 0.00)	0.005	0.01	(− 0.05, 0.08)	0.697	0.01	(− 0.04, 0.06)	0.729
LBD	1.34	(0.61, 2.94)	0.464	0.01	(− 0.02, 0.03)	0.622	0.03	(− 0.02, 0.07)	0.221	0.02	(− 0.01, 0.05)	0.262
CAA	1.71	(0.90, 3.23)	0.099	0.01	(− 0.01, 0.03)	0.283	0.02	(− 0.01, 0.04)	0.179	0.01	(− 0.01, 0.03)	0.159
Arteriolosclerosis	1.33	(0.54, 3.30)	0.536	0.01	(0.00, 0.02)	0.016	0.02	(0.00, 0.03)	0.009	0.02	(0.01, 0.03)	0.005
Atherosclerosis	1.96	(0.70, 5.49)	0.203	0.04	(− 0.02, 0.10)	0.183	0.03	(− 0.04, 0.11)	0.348	0.04	(− 0.03, 0.10)	0.275
ARTAG	2.72	(1.30, 5.69)	0.008	0.01	(− 0.01, 0.03)	0.388	0.02	(− 0.01, 0.04)	0.153	0.01	(− 0.01, 0.03)	0.173

*OR* odds ratio, *CI* confidence interval, *p* *p* value, *beta* coefficient from linear regression model

The first three columns present results from logistic regressions, where, in each regression, the outcome was the binary HS-A variable and the regressors were the pathology on the corresponding row and three baseline covariates [age at death, sex, and education (college or above vs. otherwise)]. For example, the first three cells on the first row show that the OR of having HS-A between those with ADNC and those without ADNC was 1.28, 95% CI (0.62, 2.62), and *p* value = 0.502, after adjusting for the three baseline covariates. The remaining three-column groups present results from linear regressions (where heteroskedasticity-consistent, i.e., sandwich, standard errors were used), where in each regression, the outcome was the corresponding quantitative HS-A variable and the regressors were the pathology on the corresponding row and three baseline covariates. For example, the 4th–6th cells on the first row show that the average HS-A proportion in subiculum was 0.01 higher among those with ADNC, with 95% CI (− 0.01, 0.02) and *p* value = 0.201, after adjusting for the three baseline covariates. Atherosclerosis information was missing in three individuals in the full cohort and were removed from the corresponding regression

### Hippocampal sclerosis of aging and cognition

In the full cohort, dementia was present in 44% of the individuals based on consensus conference diagnoses. Among the cognitive domains, impairment in memory was the most frequent (55%), followed by impairment in executive function (42%), orientation (37%), language (31%), calculations (20%), visuospatial function (20%), and constructions (8%) (Table 5).

**Table 5:** Co-occurrence of hippocampal sclerosis of aging and cognitive domain deficits

	Full cohort *n* = 409	Without HS-A *n* = 361	With HS-A *n* = 48	Hemisphere affected			Subfield affected		
				Right only *n* = 8	Left only *n* = 33	Bilateral *n* = 3	Subic. only *n* = 4	CA1 only *n* = 32	Both *n* = 8
Dementia	178 (43.7%)	146 (40.7%)	32 (66.7%)	2 (25%)	25 (75.8%)	3 (100%)	3 (75%)	19 (59.4%)	8 (100%)
Memory	225 (55.4%)	189 (52.8%)	36 (75%)	3 (37.5%)	27 (81.8%)	3 (100%)	3 (75%)	22 (68.8%)	8 (100%)
Language	127 (31.2%)	107 (29.8%)	20 (41.7%)	0 (0%)	15 (45.5%)	3 (100%)	2 (50%)	10 (31.2%)	6 (75%)
Executive function	165 (42%)	143 (41.3%)	22 (46.8%)	3 (37.5%)	17 (53.1%)	2 (66.7%)	3 (75%)	13 (41.9%)	6 (75%)
Visuospatial	78 (19.9%)	64 (18.6%)	14 (29.8%)	0 (0%)	11 (34.4%)	3 (100%)	0 (0%)	9 (28.1%)	5 (62.5%)
Constructions	32 (8.4%)	27 (8%)	5 (11.9%)	0 (0%)	4 (13.8%)	1 (50%)	0 (0%)	3 (10.7%)	2 (28.6%)
Calculations	79 (20.2%)	63 (18.3%)	16 (34%)	1 (12.5%)	10 (31.2%)	3 (100%)	2 (50%)	7 (21.9%)	5 (71.4%)
Orientation	149 (36.8%)	118 (33.1%)	31 (64.6%)	1 (12.5%)	25 (75.8%)	3 (100%)	2 (50%)	19 (59.4%)	8 (100%)

In the full cohort, there were individuals with missing information on dementia (*n* = 2), memory (*n* = 3), language (*n* = 2), executive function (*n* = 16), visuospatial function (*n* = 18), constructions (*n* = 30), calculations (*n* = 17), and orientation (*n* = 4). Each proportion was calculated based on the corresponding subsample without missingness

In the HS-A cohort (*n* = 48), dementia was more prevalent (67%) compared to the full cohort. Among the cognitive domains, impairment in memory was the most prevalent (75%). Notably, in those with HS-A, impairment in orientation became the second most prevalent domain (65%), followed by executive function (47%), language (42%), calculations (34%), visuospatial function (30%), and constructions (12%) (Table 5).

In the full cohort, binary HS-A (presence/absence) was significantly associated with the presence of dementia (OR = 2.77, CI 1.46–5.28, *p* = 0.002), and with impairments in memory (OR = 2.60, CI 1.30–5.18, *p* = 0.007), calculations (OR = 2.16, CI 1.09–4.28, *p* = 0.027), and orientation (OR = 3.56, CI 1.88–6.76, *p* < 0.001), with different associations for involvement of CA1 vs. subiculum (Table 6).

**Table 6:** Associations between each cognition variable and (i) binary HS-A, (ii) HS-A proportion in subiculum and CA1 combined, (iii) HS-A proportion in subiculum, and (iv) HS-A proportion in CA1 from logistic regression on the full cohort

Outcome	HS-A binary			HS-A proportion in subiculum and CA1			HS-A proportion in subiculum			HS-A proportion in CA1		
	OR	95% CI	*p* value	OR*	95% CI*	*p* value	OR*	95% CI*	*p* value	OR*	95% CI*	*p* value
Dementia	2.77	(1.46, 5.28)	0.002	2.18	(1.20, 3.96)	0.011	2.29	(1.00, 5.25)	0.050	1.65	(1.17, 2.34)	0.005
Memory	2.60	(1.30, 5.18)	0.007	1.98	(1.08, 3.64)	0.028	1.94	(0.89, 4.24)	0.095	1.55	(1.09, 2.20)	0.016
Language	1.56	(0.84, 2.93)	0.162	1.33	(1.05, 1.68)	0.018	1.38	(1.01, 1.90)	0.044	1.26	(1.05, 1.51)	0.013
Executive function	1.19	(0.64, 2.20)	0.586	1.18	(0.95, 1.48)	0.131	1.48	(0.98, 2.23)	0.061	1.06	(0.90, 1.25)	0.506
Visuospatial	1.85	(0.93, 3.69)	0.080	1.37	(1.10, 1.71)	0.006	1.58	(1.11, 2.25)	0.012	1.29	(1.09, 1.54)	0.004
Constructions	1.40	(0.50, 3.91)	0.521	1.12	(0.83, 1.51)	0.471	1.26	(0.98, 1.61)	0.067	1.14	(0.91, 1.43)	0.245
Calculations	2.16	(1.09, 4.28)	0.027	1.46	(1.13, 1.87)	0.003	1.81	(1.13, 2.90)	0.013	1.32	(1.09, 1.59)	0.004
Orientation	3.56	(1.88, 6.76)	< 0.001	2.61	(1.33, 5.12)	0.005	2.01	(1.06, 3.82)	0.033	1.81	(1.25, 2.63)	0.002

*OR* odds ratio, *CI* confidence interval, *p* *p* value, OR* odds ratio in the outcome when the corresponding HS-A proportion increases by 10 percentage points (i.e., when HS-A proportion changes from, for example, 10% to 20% or 20% to 30%), CI* confidence interval (for OR*)

We also examined the association between cognition and the proportion of HS-A in different subregions of the hippocampus. Similar to binary HS-A, we found significant associations with the presence of dementia (OR = 2.18, CI 1.20–3.96, *p* = 0.011), and with deficits in memory (OR = 1.98, CI 1.08–3.64, *p* = 0.028) and, calculations (OR = 1.46, CI 1.13–1.87, *p* = 0.003), and orientation (OR = 2.61, CI 1.33–5.12, *p* = 0.005) domains. However, with the quantitative HS-A measures, we also found significant associations with the domains of language (OR = 1.33, CI 1.05–1.68, *p* = 0.018) and visuospatial (OR = 1.37, CI 1.10–1.71, *p* = 0.006). These associations varied by involvement of CA1 vs. subiculum (Table 6).

### Hippocampal sclerosis of aging and medical history

The most prevalent medical condition in the whole population was HTN (59.4%), followed by heart disease (58.9%), osteoarthritis (52.3%), macular degeneration (39.6%), high cholesterol (35%), thyroid disease (33.3%), depression (22%), anxiety (13.9%), COPD (8.3%), and rheumatological disease (7.8%). The prevalence of these diseases was similar in those with HS-A and those without, and there were no statistically significant associations between HS-A and medical histories.

### Hippocampal sclerosis of aging and LATE-NC

To better understand the association between HS-A and LATE-NC, we further stratified these two variables. In the first set of analyses, we stratified the HS-A cohort by LATE-NC status [i.e., HS-A(+)LATE-NC(+) and HS-A(+)LATE-NC(−)]; for the second set of analyses, we stratified the LATE-NC cohort by HS-A status [i.e., LATE-NC(+)HS(+) and LATE-NC(+)HS(−)].

When stratifying the HS-A cohort by LATE-NC status, even though we found no associations with other co-occurring neuropathological changes, there were associations between HS-A(+)LATE-NC(+) and the presence of dementia (OR = 9.31, CI 2.12–40.88, *p* = 0.003) and with deficits in the visuospatial (OR = 9.57, CI 1.36–67.44, *p* = 0.023), calculations (OR = 5.74, CI 1.06–31.11, *p* = 0.043), and orientation domains (OR = 4.74, CI 1.23–18.23, *p* = 0.024) (Table 7). Furthermore, when analyzing associations with medical history, we found an association between HS-A(+)LATE-NC(+) and osteoarthritis (OR = 0.14, CI 0.03–0.66, *p* = 0.013; data not shown). Notably, there were no associations between HS-A and seizures in either LATE(+) or LATE(−) cases. Moreover, when comparing the proportion of HS-A in each subregion by presence/absence of LATE-NC, we found that on average, those individuals with HS-A(+)LATE-NC(+) had larger HS-A lesions than those HS-A(+)LATE-NC(−). These differences were statistically significant in subiculum-B (*p* = 0.034), CA1-A (*p* = 0.009), CA1-B (*p* = 0.012), and when considering both subiculum and CA1 combined (*p* = 0.003) (Table 8).

**Table 7:** Co-occurrence of LATE-NC and other neuropathological changes and cognitive domain deficits among the HS-A cohort further stratified by LATE-NC status

	HS-A(+) cohort			
	Full HS-A(+) cohort *n* = 48	LATE(+) *n* = 31	LATE(−) *n* = 17	*p* value
Age at death (years)				0.080
Mean (SD)	98.1 (3.7)	98.8 (3.5)	96.9 (3.8)	
Min, max	92.1, 106.2	92.1, 106.2	92.1, 105.6	
Sex				0.384
Female	36 (75%)	25 (80.6%)	11 (64.7%)	
Male	12 (25%)	6 (19.4%)	6 (35.3%)	
Education				0.386
Below college	28 (58.3%)	20 (64.5%)	8 (47.1%)	
College and above	20 (41.7%)	11 (35.5%)	9 (52.9%)	
Neuropathologies				
ADNC	37 (77.1%)	27 (87.1%)	10 (58.8%)	0.073
MVL	2 (4.2%)	1 (3.2%)	1 (5.9%)	0.719
LBD	9 (18.8%)	6 (19.4%)	3 (17.6%)	0.915
CAA	32 (66.7%)	22 (71%)	10 (58.8%)	0.088
Arteriolosclerosis	42 (87.5%)	29 (93.5%)	13 (76.5%)	0.347
Atherosclerosis	5 (10.6%)	4 (13.3%)	1 (5.9%)	0.504
ARTAG	38 (79.2%)	25 (80.6%)	13 (76.5%)	0.859
Cognitive domains				
Dementia	32 (66.7%)	26 (83.9%)	6 (35.3%)	0.003
Memory	36 (75%)	26 (83.9%)	10 (58.8%)	0.075
Language	20 (41.7%)	16 (51.6%)	4 (23.5%)	0.127
Executive function	22 (46.8%)	17 (54.8%)	5 (31.2%)	0.216
Visuospatial	14 (29.8%)	12 (38.7%)	2 (12.5%)	0.023
Constructions	5 (11.9%)	4 (13.8%)	1 (7.7%)	0.804
Calculations	16 (34%)	14 (46.7%)	2 (11.8%)	0.043
Orientation	31 (64.6%)	24 (77.4%)	7 (41.2%)	0.024

*p* values were obtained from *t* tests (for age at death) or Chi-squared tests (for sex and education) comparing HS-A(+)LATE-NC(+) vs HS-A(+)LATE-NC(−). Furthermore, *p* values for the associations between binary LATE-NC and co-occurring neuropathological changes were obtained from separate logistic regressions on the HS-A cohort. In each regression, the outcome was the binary LATE-NC variable and the regressors were the pathology on the corresponding row and three baseline covariates [age at death, sex, and education (college or above vs. otherwise)]. The *p* values for the associations between each cognitive variable and LATE-NC were obtained from separate logistic regressions on the HS-A cohort. In each regression, the outcome was the binary cognition variable on the corresponding row, and the regressors were the LATE-NC (binary) and three baseline covariates [age at death, sex, and education (college or above vs. otherwise)]

In each logistic regression, the outcome was one of the cognition variables and the regressors were one of the HS-A variables and three baseline covariates [age at death, sex, and education (college or above vs. otherwise)]. For example, the first three cells on the first row show that the OR of dementia between those with HS-A and those without HS-A is 2.77, 95% CI (1.46, 5.28), and *p* value = 0.002, after adjusting for the three baseline covariates. Furthermore, the 4th–6th cells on the first row show that the odds of dementia increased by 2.18-fold when the HS-A proportion in both subiculum and CA1 combined increased by 10 percentage points, 95% CI (1.20, 3.96), and *p* value = 0.001, after adjusting for the three baseline covariates

**Table 8:** Average proportions of hippocampal sclerosis of aging in each subregion stratified by LATE-NC status, among the HS-A cohort

	Average HS-A proportion (%) among HS-A(+)LATE-NC(+)	Average HS-A proportion (%) among HS-A(+)LATE-NC(−)	Difference in proportion	*t* value	*df*	*p* value
Subiculum						
A	7.1	5.0	− 2.1	− 0.30	38	0.766
B	13.7	0.0	− 13.7	− 2.24	27	0.034
C	25.9	10.7	− 15.2	− 1.41	40	0.165
CA1						
A	36.5	6.3	− 30.2	− 2.75	41	0.009
B	48.0	20.5	− 27.5	− 2.63	40	0.012
C	19.2	9.9	− 9.3	− 1.73	38	0.091
Subiculum and CA1 combined	29.1	9.7	− 19.4	− 3.22	36	0.003

The first column represents the average of HS-A proportion in participants with HS-A(+)LATE-NC(+) in the corresponding subregion (*n* = 31). For example, the first entry 7.1 means that among those with HS-A(+)LATE-NC(+), the average HS-A proportion in subiculum-A is 7.1%. The second column is the average of HS-A proportion participants with HS-A(+)LATE-NC(−) in the corresponding subregion, among those with HS-A(+)LATE-NC(−) (*n* = 17). Degrees of freedom (*df*) and *p* value are from Welch’s *t* test that accounts for unequal variances

We then stratified the LATE-NC cohort by HS-A status (i.e., binary and quantitative; Table 9). Interestingly, among five individuals with HS-A(−)LATE-NC(+) who had pure LATE-NC, four had dementia. When assessing co-occurrence of neuropathological changes, we found that the odds of having HS-A were 2.56 times higher in those with CAA (CI 1.05–6.27, *p* = 0.039). Furthermore, we found that the odds of dementia were 4.01 (CI 1.42–11.34, *p* = 0.009), 2.86 for deficits in calculations (CI 1.18–6.91, *p* = 0.020), and 3.34 for deficits in orientation (CI 1.31–8.50, *p* = 0.011) between those with HS-A and those without HS-A. There were no associations between medical history and HS-A.

**Table 9:** Co-occurrence of HS-A and other neuropathological changes and cognitive domain deficits among the LATE-NC cohort

	LATE cohort *n* = 149	LATE(+)HS(+) *n* = 31	LATE(+)HS(−) *n* = 118	*p* value
Age at death (years)				0.325
Mean (SD)	98 (3.7)	98.8 (3.5)	97.8 (3.8)	
Min, Max	90.8, 110.6	92.1, 106.2	90.8, 110.6	
Sex				0.469
Female	104 (69.8%)	25 (80.6%)	79 (66.9%)	
Male	45 (30.2%)	6 (19.4%)	39 (33.1%)	
Education				0.244
Below college	70 (47%)	20 (64.5%)	50 (42.4%)	
College and above	79 (53%)	11 (35.5%)	68 (57.6%)	
LATE-NC stage				
Amygdala	22 (14.8%)	7 (22.6%)	15 (12.7%)	
Hippocampus	115 (77.2%)	18 (58.1%)	97 (82.2%)	
Neocortex	12 (8%)	6 (19.3%)	6 (5.1%)	
Neuropathologies				
ADNC	119 (79.9%)	27 (87.1%)	92 (78%)	0.224
MVL	6 (4%)	1 (3.2%)	5 (4.2%)	0.819
LBD	26 (17.4%)	6 (19.4%)	20 (16.9%)	0.645
CAA	85 (57%)	22 (71%)	63 (53.4%)	0.039
Arteriolosclerosis	126 (84.6%)	29 (93.5%)	97 (82.2%)	0.297
Atherosclerosis	12 (8.2%)	4 (13.3%)	8 (6.8%)	0.181
ARTAG	111 (74.5%)	25 (80.6%)	86 (72.9%)	0.591
Cognitive domains				
Dementia	90 (60.4%)	26 (83.9%)	64 (54.2%)	0.009
Memory	107 (71.8%)	26 (83.9%)	81 (68.6%)	0.106
Language	64 (43%)	16 (51.6%)	48 (40.7%)	0.400
Executive function	67 (47.5%)	17 (54.8%)	50 (45.5%)	0.605
Visuospatial	40 (28.4%)	12 (38.7%)	28 (25.5%)	0.114
Constructions	15 (10.9%)	4 (13.8%)	11 (10.1%)	0.823
Calculations	37 (26.1%)	14 (46.7%)	23 (20.5%)	0.020
Orientation	80 (53.7%)	24 (77.4%)	56 (47.5%)	0.011

*p* values were obtained from *t* tests (for age at death) or Chi-squared tests (for sex and education) comparing those with HS-A and those without HS-A in the full cohort. *p* values for neuropathologies were obtained from logistic regressions, where in each regression, the outcome was the binary HS-A variable and the regressors were the pathology on the corresponding row and three baseline covariates [age at death, sex, and education (college or above vs. otherwise)]. *p* values for cognitive domains were obtained from logistic regressions where the outcome was one of the cognition variables and the regressors were one of the HS-A variables and three baseline covariates [age at death, sex, and education (college or above vs. otherwise)]

Atherosclerosis information was missing in two individuals in the LATE-NC cohort; hence, the corresponding proportions were calculated after removing these individuals

## Discussion

Hippocampal sclerosis of aging (HS-A) is a neuropathological change that is relatively common in the elderly population and is strongly associated with dementia in the oldest-old [17, 29, 30, 38, 39, 45]. For this reason, furthering our understanding of HS-A in this population is fundamental. In this study, we analyzed the prevalence and distribution of HS-A in 409 participants of *The 90*+ *study*, a prospective clinicopathological study of the oldest-old. We found that HS-A was present in 12% of the cohort, was almost always unilateral (93%), and consistently focal, always sparing some parts of CA1 or subiculum. We found HS-A was far more frequent in CA1 region (89%) than subiculum (27%) and that participants with HS-A did not have evidence of this lesion in hippocampal subfields other than CA1. Involvement of the mid-section of CA1 subfield was more frequent than other subregions with 82% of those with HS-A presenting with involvement of this subregion. Furthermore, we found that in 18% of those with HS-A, these pathological lesions affected both CA1 and subiculum. Left side lesions were more prevalent (82%) and larger than those in the right side. Moreover, dementia and impairment of cognitive domains were more frequent in left-sided HS-A. In terms of neuropathological associations, we found that LATE-NC and ARTAG were the only neuropathological changes associated with HS-A when the diagnosis of HS-A was assigned traditionally as a binary diagnosis. Our novel quantitative measurement of HS-A, however, revealed additional association of HS-A in both subiculum and CA1 subfield (both separately and combined) with arteriolosclerosis. We also identified a novel inverse association of subicular HS-A with MVL. From a cognitive standpoint, we found that traditional binary HS-A measures were associated with outcomes of dementia as well as impairment in memory, orientation, and calculation with different associations for involvement of CA1 vs. subiculum. Our novel quantitative approach showed additional associations between the proportion of HS-A in subiculum and CA1 (both separately and combined) impairments in language and visuospatial domains. Moreover, memory impairment was associated with the proportion of HS-A in CA1 and when combined in subiculum and CA1, but not in subiculum only.

Similar prevalence rates of HS-A in individuals over 85 years old have been previously reported [31, 38]. Other studies, however, have reported a wide range in prevalence, from 0.4% up to 25% [2, 60]. These discrepancies could be due to differences in histopathologic criteria (i.e., HS-A being defined as neuronal loss in both subiculum *and* CA1 [2, 14, 46], in subiculum *and/or* CA1 [6, 16, 28, 31, 36, 39], or within CA1 only [7, 23]). In addition, the use of different methodologies may also contribute to the discrepancies. For instance, the low prevalence of 0.4% reported by Ala et al.is the result of diagnosing HS-A when it was a standalone lesion. Given the very high prevalence of co-occurring pathological changes in the oldest-old, this criterion likely led to exclusion of most participants with HS-A [2]. Similarly, Kero et al. reported a low prevalence of pure HS-A (2%), compared to a 16% prevalence of HS-A when co-morbid neuropathological changes were allowed [30]. Moreover, it is a common practice in neuropathology laboratories to freeze half of the brain after removal and only examine one hemisphere for presence of pathologic changes. Given the high prevalence of unilateral HS-A, only examining one hemisphere leads to an underestimation of the prevalence of this disease [60].

In terms of laterality, we found that HS-A was more frequent in the left hemisphere (82%) than in the right (25%), and that bilateral HS-A was the least frequent (7%). Similar to our findings, Probst et al*.* reported unilateral left-sided HS-A to be the most frequent (5/10) although they had a higher rate of bilateral HS-A (3/10) that was more frequent than unilateral right-sided (2/10) HS-A [47]. In contrast, other studies have described a higher prevalence of bilateral HS-A [31, 60]. In one of these studies, bilateral HS-A was present in 45% (14/31) of individuals, followed by left- (10/31; 32%) and right-sided HS-A (8/31; 23%) [60]. Another study with a larger sample size reported a higher prevalence of bilateral HS-A (64/106; 60.4%), followed by left- (26/106; 24.5%) and right-sided HS-A (16/106; 15.1%) [38]. Similarly, Kero et al. found bilateral HS-A in 24/47 (51%) of individuals and unilateral HS-A in 23/47 (49%), with left-sided HS-A being more common than right-sided [30]. In addition to differences between cohorts and the characteristics of participants, lack of consensus criteria for histopathologic diagnosis of HS-A in the presence of other pathologic lesions, including Alzheimer’s disease, is an alternative potential explanation for the observed discrepancies. These variable results from different cohorts highlight the need for developing consensus guidelines for the pathologic evaluation of HS-A [41].

According to current operational recommendations, HS-A may be defined as neuronal loss in the CA1 and/or subiculum. We found that the distribution of HS-A varied between these two subfields. In agreement with Hokkanen et al. [24], we found that HS-A was far more frequent in the CA1 subfield of hippocampus (89%) than subiculum (27%). Moreover, involvement of both subiculum and CA1 was found in eight (18%) of the individuals. In contrast, and in agreement with previous reports [60], we found that CA2 and CA3 were spared of HS-A. The extent and distribution of HS-A varied between the subregions of subiculum and CA1. In subiculum, HS-A mostly affected subiculum-C (the portion adjacent to CA1) alone (6/12), followed by overlapping subiculum-B and -C (3/12), and involvement of the whole subiculum (2/12). In CA1 subfield, however, involvement of multiple subregions was the most common observation (17/39) followed by involvement of mid portion of CA1 alone (12/39), and solitary involvement of the subregion adjacent to CA2 subfield (9/39). Moreover, we found 8 out of 12 individuals with HS-A in subiculum had overlapping HS-A in CA1. It is noteworthy that seven of these individuals (87%) had concomitant LATE-NC. In contrast, of the remaining five individuals with HS-A in subiculum only, only two of them were LATE-NC positive. In a previous study, Hokkanen et al. assessed the prevalence of TDP-43 pathology in different subfields of the hippocampus. In this study, TDP-43 pathology was present in all the individuals with HS-A, and TDP-43 pathological inclusions were most commonly found within the CA1 subfield [24].

Previous studies have reported the association of HS-A with co-occurring neuropathological changes including LATE-NC, vascular disease, and ADNC [5, 17, 31, 38, 47, 60]. In our study, the associations between HS-A and other co-occurring neuropathological changes differed when we used traditional binary diagnosis of HS-A (presence/absence) vs. our novel quantitative measures. Our analysis based on binary presence of HS-A, suggested an association between HS-A and LATE-NC and ARTAG. Our novel quantitative analyses, on the other hand, re-demonstrated the association between HS-A (CA1/subiculum/combined) and LATE-NC but did not replicate the ARTAG association seen with the binary measure. Quantitative analysis, however, identified a new association between HS-A (CA1/subiculum/combined) and arteriolosclerosis. These findings suggest that the use of quantitative analyses offers an insight that cannot be gained from traditional binary classification. Concomitant LATE-NC was present in around two thirds (64%) of individuals who had HS-A at autopsy and was significantly associated with HS-A in both binary and quantitative analyses. The association between HS-A and LATE-NC has been the most consistent finding of previous studies and LATE-NC has been reported in 50–90% of the individuals with HS-A [33, 38, 60]. Even though these are independent pathologies, a recent study has proposed that HS-A progression follows a similar distribution pattern as that of LATE-NC [42]. Some researchers consider pathologic TDP-43 inclusions a pre-requisite for development of HS-A. We found, however, that more than one third of those with HS-A were negative for pathologic TDP-43 inclusions.

To explore the association between HS-A and LATE-NC further, we first stratified those with HS-A by LATE-NC status. This approach revealed that HS-A lesions are more severe in those with LATE-NC. Furthermore, we found that participants HS-A(+)LATE-NC(+) had worse cognitive scores than those HS-A(+)LATE-NC(−), particularly in the visuospatial, calculations and orientation domains. We then stratified those with LATE-NC by HS-A status and we confirmed associations with dementia and with deficits in calculations and orientation domains, suggesting that the presence of HS-A makes LATE more consequential either directly or indirectly due to more common occurrence of LATE-NC stage 3 in the HS-A(+) subset. Our findings agree with previous reports that showed that those individuals HS-A(+)LATE-NC(+) had severe impairments in personal care and orientation domains, than those without HS-A, and a tendency to present with more severe atherosclerosis and arteriolosclerosis [20]. Notably, an association between LATE-NC and arteriosclerosis has been previously reported [9] and has also been described in the oldest-old [21].

Our findings provided evidence suggesting that even though HS-A and LATE-NC are frequent comorbidities, neither of them is necessarily causative of the other and, therefore, should be treated as independent neuropathologic changes. For this reason, we propose using the term HS-A as this terminology reflects the age-related nature of this prevalent neuropathologic change and distinguishes HS-A from LATE-NC. Moreover, we propose that future characterization of HS-A should at a minimum include the laterality and spread of pathologic changes observed (i.e., whether the pathology is limited to CA1, subiculum, or involves both), and preferably include a semi-quantitative or quantitative measure of the extent of pathology in each subfield like the one used in this study. A collaborative approach to defining HS-A will greatly improve the reliability and inter-observer agreement of pathological diagnosis of HS-A which will in turn strengthen the efforts to identify in-vivo biomarkers for this important pathologic change.

Arteriolosclerosis was the most prevalent neuropathological change in this cohort. Using our quantitative method, we found that presence of arteriolosclerosis was significantly associated with proportion of HS-A in subiculum, CA1, and when HS-A was affecting both subfields. Arteriolosclerosis might provide an alternative pathway for developing HS-A. Previous studies have reported an association between the presence of arteriolosclerosis, particularly in the frontal cortex, and the development of HS-A [17, 26, 40]. Our assessments did not specify the region of arteriolosclerosis but rather assessed its severity in the most extensively involved region of cerebral cortex, and therefore, our study was not suited to investigate the association between brain region of arteriolosclerosis and HS-A.

We also found an inverse association between proportion of HS-A in subiculum and the presence of MVL. This result was mainly due to the absence of MVL in those with subicular HS-A. Due to low number of individuals with HS-A in subiculum, further studies are needed to replicate our findings.

In agreement with previous studies in the oldest-old [49], ARTAG was a highly prevalent co-occurring neuropathological change, affecting 60% of individuals in the full cohort. The presence of ARTAG was associated with HS-A when assessed using traditional binary methods. This finding was not replicated when using quantitative measures. Previous studies have reported a link between the presence of HS-A and non-AD tauopathies [4, 30]; however, it has been suggested that these associations might be less specific, as the presence of tauopathies can be caused by diverse factors, such as chronic traumatic brain injury [50]. We did not investigate the association of different subtypes of ARTAG (subpial, white matter, cortical) with HS-A, and future studies should investigate the link between HS-A and different ARTAG subtypes. Notably, a link between the presence of granular fuzzy astrocytes (GFA), a subtype of ARTAG, and argyrophilic grain disease (AGD) has been proposed [12]. In fact, it has been suggested that the presence of GFA in the limbic region may precede the formation of argyrophilic grains. AGD is a common tauopathy in the oldest-old [18] that presents with a left-side predominant asymmetric distribution [1] and which has been previously associated with severe neuronal loss in amygdala and CA1 [51], and with TDP-43 pathology [19]. Since the presence of AGD was not examined, it was not possible to investigate the associations between AGD and HS-A in this cohort.

The association between HS-A and dementia and memory impairment has been consistently reported [14, 17, 27]. Our findings aligned with this consensus. Using traditional binary measures, we found presence of HS-A to be significantly associated with dementia and impairment in memory, calculation, and orientation. Our novel quantitative approach additionally identified impairment of language and visuospatial function impairment to be related to HS-A. Investigating the specific contribution of CA1 and subiculum involvement to the impairment of cognitive domains revealed a largely comparable pattern. Involvement of CA1 (but not subiculum) was associated with impairment in memory. Involvement of both regions was associated with impairment in language, visuospatial, orientation, calculation, and orientation domains. While our study did not include a comparison of the neuropsychological profiles of HS-A and ADNC, the pattern of impairment observed in individuals with HS-A would be most consistent with a clinical diagnosis of Alzheimer’s disease. Previous studies have reported on the similarity of the clinical presentation of these two diseases [13, 14, 31, 53, 58].

A major strength of this study was the inclusion of a large number of oldest-old individuals with regular longitudinal neuropsychological assessments whose neuropathological examinations were done consistently by the same neuropathologist minimizing the risk of diagnostic bias given the relatively subjective nature of HS-A neuropathological diagnosis. Our novel neuropathological assessments added a quantitative dimension to the diagnosis of HS-A that was lacking in most of the previous studies. Lack of ethnic diversity, on the other hand, was a major limitation of our study that limits the generalizability of our findings for individuals of non-Caucasian ethnic background.

In summary, HS-A is a prevalent neuropathological finding in the aging population. The great variability of HS-A prevalence in the literature highlights the need for adopting a systematic and standardized approach for the pathological assessment of HS-A. Here, we introduced a quantitative method for HS-A analysis that identified novel associations between HS-A and other neuropathological changes and impairment in cognitive domains. In our cohort of oldest-old individuals, we found that HS-A was largely unilateral and focal, with no case involving the entirety of both CA1 and subiculum. As expected, we found HS-A in both CA1 and subiculum to be associated with LATE-NC. We also found that HS-A in both CA1 and subiculum subregions was associated with arteriolosclerosis. HS-A in our cohort was associated with a clinical presentation that would be best described as “Alzheimer’s like”. Our findings imply LATE-NC and vascular mechanisms as potentially distinct upstream mechanisms for development of HS-A and that using quantitative measurements provide an insight that cannot be gained through traditional binary measures.
